# Non-destructive diagnosis of Inflammatory Bowel Disease by near-infrared spectroscopy and aquaphotomics

**DOI:** 10.1038/s41598-024-65443-0

**Published:** 2024-07-10

**Authors:** Saba Behdad, Reza Massudi, Abbas Pakdel

**Affiliations:** 1 Department of Animal Science, College of Agriculture, Science and Research Branch, Islamic Azad University, Tehran, 14778-93855 Iran; 2 Laser and Plasma Research Institute, Shahid Beheshti University, Tehran, 19839-69411 Iran; 3 Department of Animal Science, College of Agriculture, Isfahan University Of Technology (IUT), Isfahan, 84156-83111 Iran

**Keywords:** Biophysics, Developmental biology, Immunology, Biomarkers, Diseases, Gastroenterology, Medical research, Pathogenesis, Optics and photonics

## Abstract

Inflammatory Bowel Disease includes Crohn's Disease and Ulcerative Colitis. Currently, diagnosing involves a series of current diagnostic methods that are invasive, time-consuming, and expensive. Near-infrared spectroscopy and aquaphotomics can detect changes in biofluids and thus have the potential to diagnose disease. This study aimed to investigate the diagnostic ability of near infrared spectroscopy and aquaphotomics for Inflammatory Bowel Disease and its types. This method used blood plasma and saliva samples absorbance spectrum and multivariate analysis with the Principal Component Analysis and, Linear Discriminant Analysis, Quadratic Discriminant Analysis, and Support Vector Machine in the range 1300–1600 nm and 12 water absorbance bands in this range, separately. In the near-infrared range, total accuracy of 100% led to the separation of the healthy group and Inflammatory Bowel Disease and then the separation of the healthy group and patients with Ulcerative Colitis and Crohn's Disease. The aquaphotomics approach was used to investigate the changes in the 12 water absorbance bands and their impact on the accuracy of the diagnostic method. Aquaphotomics also detected 100% of the mentioned samples. We achieved a fast, accurate, non-invasive method based on near-infrared spectroscopy and aquaphotomics to diagnose Inflammatory Bowel Disease and its types using blood plasma or saliva samples. The current study found that monitoring blood plasma or saliva using near-infrared spectra offers an opportunity to thoroughly investigate biofluids and changes in their water spectral patterns caused by complex physiological changes due to Inflammatory Bowel Disease and its types, and to visualize these changes using aquagram.

## Introduction

Inflammatory Bowel Disease (IBD) includes Crohn's Disease (CD) and Ulcerative Colitis (UC), which has become a global disease at the beginning of the twenty-first century. This disease is spreading with a prevalence rate of more than 0.3% in the world and an increasing incidence rate in industrialized or developing countries^1,2^, which highlights the need for research on the prevention of IBD and innovation in diagnosis and healthcare systems to manage this complex and expensive disease^3,4^.

Although the primary cause of IBD is still unknown, environmental factors, including viruses and bacteria like MAP (*Mycobacterium Avium* subspecies *Paratuberculosis*, the agent that causes Johne’s disease in domestic animals)^5^, genetic factors, and issues with the intestinal flora are known to contribute to this conditions^5^. MAP is transmitted from infected livestock, like dairy cattle to milk, meat, and water and can infect humans^5^.

Standard diagnostic methods for IBD include stool and blood tests, biopsy, sigmoidoscopy, colonoscopy, endoscopy, and eventually, CT scan and X-ray imaging with barium enema. There is no definitive cure for IBD and symptoms are managed with medication, stress reduction, and occasionally surgery. The goal is to control inflammation, eliminate nutritional problems, relieve symptoms, and reduce recurrence. The course of treatment depends on the place where the inflammation occurs, the severity of the disease, the type of disease, and the individual's response to previous treatment^6^.

Diagnostic work-up for diagnosing IBD still is complex and varies in clinical practice^7^. Early recognition along with appropriate treatment slows down the progression of the disease and reduces severe relapses, intestinal penetration, or the need for invasive surgeries. Additionally, early diagnosis helps to identify patients in the early stage of the disease and avoid unnecessary, harmful, and expensive treatment. The diagnosis of CD or UC in different societies will also lead to correct health and treatment policies^8^.

Near-infrared (NIR) spectroscopy is used to detect functional groups that help identify molecules and their compounds. In this method, the NIR radiation has less energy than the visible and ultraviolet rays and only causes the rotation and bending of the bonds of the functional groups. The uses of NIR spectroscopy include the ability to recognize molecular alterations brought on by physical changes, comprehend the molecular causes of various diseases, and identify biomarkers through the spectrum that can be employed in disease diagnosis^9,10^.

The aquaphotomics approach was founded in 2005 by Professor Rumania Tsenkova^11^. Based on this science, the structure of water in the bodies of bio-organisms changes under the influence of perturbance factors, and water as a comprehensive biomolecule is a full-view mirror of the processes occurring in the body due to perturbance factors, including disease. The reaction of body water to perturbance-like disease, can be measured by observing the changes in the 12 water absorbance bands or Water Matrices Coordinates (WAMACs) in the range of 1300–1600 nm, absorbance bands attributed to specific water molecular conformations and these changes can be seen in the star chart, aquagram^12,13^. The characteristics of the 12 water absorbance bands are given in the method section.

**Figure 1:**
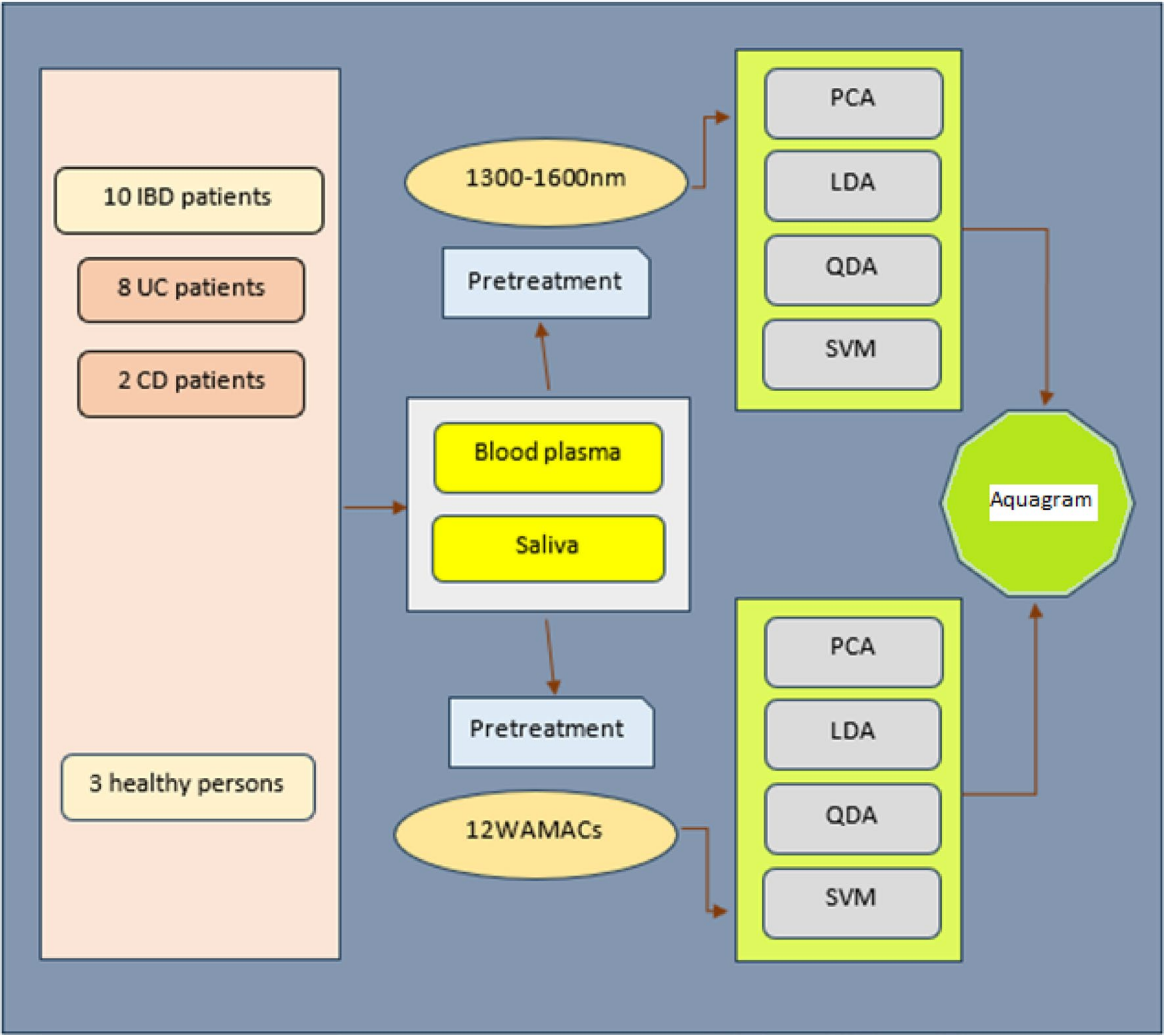
The abstract of the study “Non-destructive diagnosis of Inflammatory Bowel Disease by near-infrared spectroscopy and aquaphotomics”. *IBD* (Inflammatory Bowel Disease), *UC* (Ulcerative Colitis), *CD* (Crohn’s Disease), *WAMACs* (Water Matrices Coordinates), *PCA* (Principal Component Analysis), *LDA* (Linear Discriminate Analysis), *QDA* (Quadratic Discriminate Analysis), *SVM* (Support Vector Machine).

This study aims to develop a fast, non-destructive, and accurate method based on NIR spectroscopy and aquaphotomics approach to detect blood plasma or saliva samples of healthy people from IBD patients and then distinguish between CD and UC patients. Figure 1 shows the abstract of this study.

## Results

### Questionnaire

About the effect of MAP and IBD infection, the healthy group and patients were asked about contact with livestock, agricultural activities, non-piped water, non-pasteurized dairy products, family history, place of residence in childhood and now, and occupation. Out of a total of 10 patients with IBD, 30% had a family history, 70% now resided in cities, and 70% spent their childhood years in villages. 90% of the case group uses piped water now, and 70% used non-piped water when they were children. 50% of the case group used unpasteurized milk and dairy products during childhood and now. 20% were non-slaughterhouse meat consumers; 50% had agriculture activity; 40% were in contact with livestock; 60% were in contact with soil and manure; 10% were employed in slaughterhouses; and finally, 80% were in contact with milk and dairy product production. These results can confirm the effect of contact with contaminated livestock and its products, including milk, meat, and their products, and contaminated manure and water on IBD rate.

### Raw absorbance spectra of NIR spectroscopy

**Figure 2:**
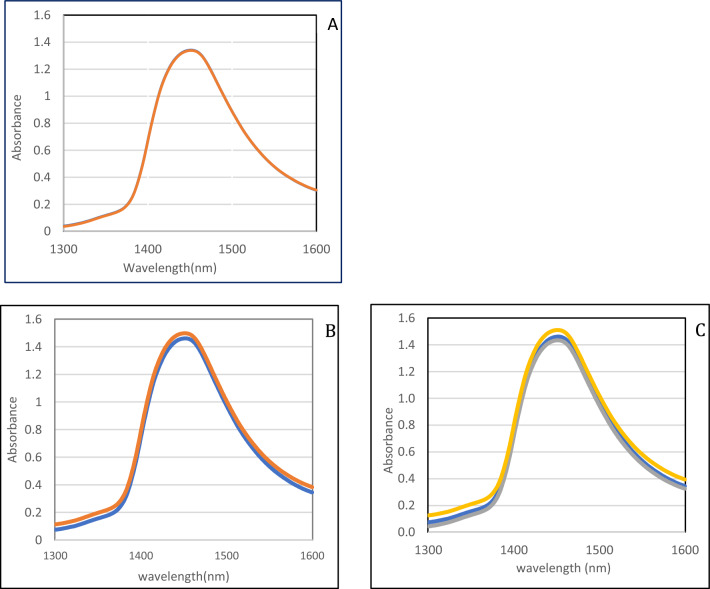
The mean spectrums. (**A**) blood plasma of the healthy group(blue) and the patients with IBD (red). (**B**) saliva data of the healthy group(blue) and the patients with IBD (red). (**C**) saliva of the healthy group (Blue), Crohn's patient(gray), and Ulcerative Colitis patient(yellow).

The highest absorption at about 1450 nm confirms that the spectrum of blood plasma and saliva is dominated by water, portion of water in plasma and saliva is 91% and 99%, respectively. In the raw spectrum of the blood plasma, no obvious differences were seen between the healthy group and IBD patients (Fig. 2A). The saliva average spectrum of IBD patients was higher than the healthy group (Fig. 2B) and CD and UC patients were lower and higher than the average spectrum of healthy people, respectively (Fig. 2C).

**Figure 3:**
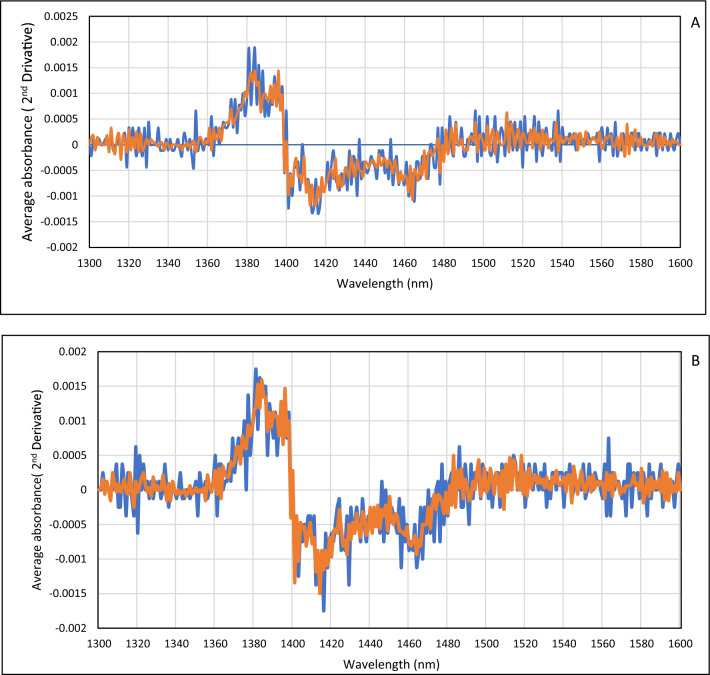
2nd derivative (calculated with a symmetric Savitzky -Golay Smoothed with 12 points) average absorbance spectra in the spectral range of 1300–1600 nm (OH first overtone) of healthy group (Blue) and patients with IBD (Red). (**A**) blood plasma (**B**) saliva.

Using the average absorbance spectra of the second derivative of the data, the difference between the two groups of healthy and IBD was evident in all wavelengths, especially in the C4 to C10 water bands (Fig. 3).

Except for a small number of wavelengths in the region of 1300–1600 nm, the difference spectrum reveals a discernible difference between the average of IBD patients and the healthy group. Most of these differences are in the range of 12 water absorbance bands and the rest of the cases also have significant differences (Fig. 3). Then, the chemometrics was performed using The Unscrambler® X software (Version 10.3. CAMO SOFTWARE, Oslo, NORWAY).

### Principal component analysis (PCA)

**Figure 4:**
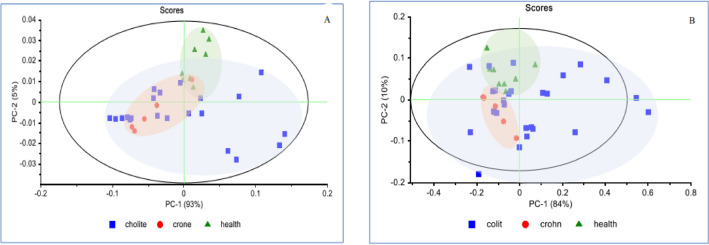
PCA Scores plot- scattering of spectral data based on SNV pretreatment. (**A**) blood plasma. **(B)** saliva. healthy group(green), Crohn's Disease patients (red), and Ulcerative Colitis patients (blue).

The PCA is an unsupervised multivariate analysis that can reduce the dimensionality of data sets, explain variation in the data by ignoring data labels, detect patterns in spectral behavior, and find excluded data. The PCA results are present in the “Scores” and “Loading” diagrams. Using the raw data from the “Scores” diagram in blood plasma, PC1 50%, PC2 43%, PC3 4%, and PC4 2% described the variance between the data (Fig. 4A). Using Standard Normal Variate (SNV) pretreatment, four principal components (PCs) described 100% of the variance between the data (PC1, 93%; PC2 with 5%; and PC3 and PC4 with 1% each). In the space of PC1–PC2, the healthy group and IBD patients, including CDs and UCs, were separated from each other.

In saliva, based on the “Scores” plot in the raw data, PC1 was 99% and PC2 was 1%, and using SNV pretreatment, six PCs described 100% of the variance between the data (PC1 was 84%, PC2 was 10%, PC3 was 3%, and PC4 and PC6 were 1% each) (Fig. 4B).

**Figure 5:**
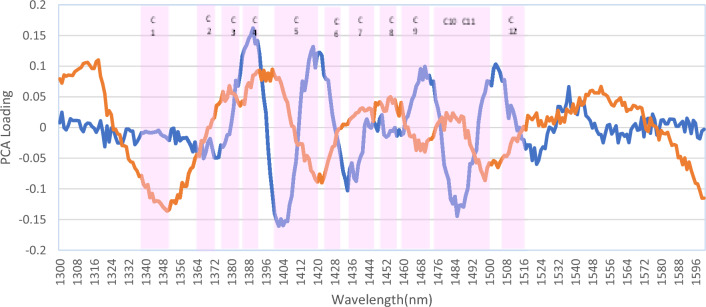
PCA “Loading” plot for blood plasma (PC4) (Blue) and saliva (PC6) (Red). The pink bands are 12 water absorbance bands in the NIR range.

Based on the “Loading” diagram of PC4 of blood plasma and PC6 of saliva (Fig. 5), showed variable wavelengths related to the location of water absorbance bands, which can explain the observed pattern of changes in IBD. By comparing these PCs, more than 12 peaks were identified in the blood plasma “Loading” diagram, and these peaks were larger than saliva peaks and had an inverse relationship in most cases. Important wavelengths were observed at 1390, 1402, 1418, 1422, 1434, 1438, 1450, 1485, 1503, 1522, 1537, and 1553 nm.

### Linear discriminant analysis (LDA)

LDA is the simplest classification method that supervises dimensionality reduction techniques, classifies data simultaneously, and focuses on finding a feature subspace that maximizes group separability. The variables with binary or multiclass labels are the target of LDA^14^.

LDA, in the range 1300–1600 nm, separates the plasma samples of healthy people and patients with IBD by raw data using internal and external validation methods with accuracy, sensitivity, and specificity of 100%. Using Normalized, and smoothed pretreatments, achieved the same results.

In the range of 12 water absorbance bands, the separation of plasma samples of healthy people and patients with IBD in the internal and external validation in SNV, MSC, and Normalized pretreatments reached 100% total accuracy, sensitivity, and specificity, which shows that all the effective factors have manifested in their water absorbance bands (Table 1).

**Table 1:** The results of supervised methods (LDA, QDA and SVM) of the blood plasma spectra of healthy, IBD, CD and UC patient by NIR spectroscopy in 1300–1600 nm and aquaphotomics approach in 12 WAMACs.

Model	Range	Contents	Blood plasma—predicted model		
			Total-Full validation (%)	Calibration-Full validation (%)	Test-Cross validation (%)
LDA	1300–1600 (nm)	Healthy	100	100 100	100
		IBD patient	100	94 88	100
		Total accuracy	100	95.6 91	100
		Pretreatment	Raw data-All pretreatment	spectro scopic SNV-MSC-Normalized	Raw data-Smoothed- Normalized
		PC	7	4	4
	12 water absorbance bands	Healthy	100	100	100
		IBD	100	87	100
		Total accuracy	100	90.4	100
		Pretreatment	All pretreatment	Raw data—Smoothed	Normalized-SNV-MSC
		PC	7	4	4
QDA	1300–1600 (nm)	Healthy	100	100	100
		IBD	100	100	100
		Total accuracy	100	100	100
		Pretreatment	Raw data- All pretreatment	Normalized	SNV-MSC
		PC	4	4	4
	12 water absorbance bands	Healthy	100	100	66
		IBD	100	100	100
		Total accuracy	100	100	92
		Pretreatment	Normalized-SNV-MSC	Raw data-All pretreatment	Normalized
		PC	4	4	4
	1300–1600 (nm)	Healthy	100	100	100
		Ulcerative Colitis	100	100	100
		Crohn's Disease	100	100	0
		Total accuracy	100	100	84.6
		Pretreatment	Raw data-All pretreatment	Normalized	SNV-MSC
		PC	4	4	4
	12 water absorbance bands	Healthy	100	100	100
		Ulcerative Colitis	100	100	100
		Crohn's Disease	100	100	0
		Total accuracy	100	100	84.6
		Pretreatment	SNV	Raw data- Smoothed	SNV-MSC
		PC	4	4	4
SVM	1300–1600 (nm)	IBD	100	100	100
		Healthy	100	100	100
		Pretreatment	Raw Data—All pretreatment	Raw Data -All pretreatment	Raw Data-All pretreatment
		Total Accuracy	100	100	100
		R2 -RMSEC	99–0.04	99–0.04	99–0.04
	12 water absorbance bands	IBD	100	100	100
		Healthy	100	100	100
		Pretreatment	Raw Data—All pretreatment	Raw Data -All pretreatment	SNV-MSC-Smoothed
		Total accuracy	100	100	100
		R2 -RMSEC	99–0.04	99–0.04	99–0.04
	1300–1600 (nm)	Healthy	100	100	100
		Ulcerative Colitis	100	100	100
		Crohn's Disease	100	100	100
		Pretreatment	Raw Data -All pretreatment	Raw Data -All pretreatment	Raw Data-Smoothed
		Total accuracy	100	100	100
		R2 -RMSEC	98–0.09	98–0.09	98–0.09
	12 water absorbance bands	Healthy	100	100	100
		Ulcerative Colitis	100	100	100
		Crohn's Disease	100	100	50
		Pretreatment	Raw Data -All pretreatment	Raw Data -All pretreatment	MSC
		Total accuracy	100	100	92.3
		R2 -RMSEC	98–0.09	98–0.09	98–0.09

LDA, in the range 1300–1600 nm, separates the saliva samples of two groups of healthy and IBD patients by SNV and MSC pretreatment, in internal validation reaching 93.7% sensitivity and 100% specificity. In external validation, Normalized and MSC pretreatments reached 100% total accuracy, sensitivity, and specificity (Table 2).

**Table 2:** The results of supervised methods (LDA, QDA and SVM) of the saliva spectra of healthy, IBD, CD and UC patient by NIR spectroscopy in 1300–1600 nm and Aquaphotomics approach in 12 WAMACs.

Model	Range	Contents	Saliva—Predicted model		
			Total—Full validation (%)	Calibration—Full validation (%)	Test-Cross-validation (%)
LDA	1300–1600 (nm)	Healthy	100	100	100
		IBD	93.7	92	100
		Total accuracy	95	94	100
		Pretreatment	SNV -MSC	Normalized	Normalized—MSC
		PC	7	7	4
	12 water absorbance bands	Healthy	100	100	100
		IBD	93.7	92	62.5
		Total accuracy	95	94	70
		Pretreatment	SNV -MSC	Raw Data- Smoothed	Raw Data- All pretreatment
		PC	7	7	6
QDA	1300–1600 (nm)	Healthy	100	100	100
		IBD	100	100	100
		Total accuracy	100	100	100
		Pretreatment	Normalized-SNV-MSC	Raw Data- all pretreatment	SNV- MSC
		PC	4,4,5	4,4,5	4
	12 water absorbance bands	Healthy	100	100	50
		IBD	100	100	100
		Total accuracy	100	100	90
		Pretreatment	Raw Data- all pretreatment	All pretreatment	Smoothed—Normalized
		PC	6, 4,5	4,5	4
	1300–1600 (nm)	Healthy	100	100	100
		Ulcerative colitis	100	90.4–95.2	100
		Crohn's disease	100	100	0
		Total accuracy	100	93–96	80
		Pretreatment	SNV -MSC	MSC -Normalized	MSC
		PC	4,5	4	4
	12 water absorbance bands	Healthy	100	100	100
		Ulcerative colitis	100	90.4–95.2	83.3
		Crohn's disease	100	100	0
		Total accuracy	100	93–96	70
		Pretreatment	Raw Data	MSC -Normalized	MSC
		PC	6	5	4
SVM	1300–1600 (nm)	IBD	100	100	100
		Healthy	100	100	100
		Pretreatment	Raw Data- all pretreatment	Raw Data- all pretreatment	SNV-Normalized
		Total accuracy	100	100	100
		R2-RMSEC	99–0.04	99–0.04	99–0.04
	12 water absorbance bands	IBD	100	100	100
		Healthy	100	100	0
		Pretreatment	Raw Data- all pretreatment	Raw Data- all pretreatment	Raw Data- Smoothed—Normalized
		Total accuracy	100	100	80
		R2 -RMSEC	99–0.04	99–0.04	98–0.09
	1300–1600 (nm)	Healthy	100	100	100
		Ulcerative Colitis	100	100	83
		Crohn's Disease	100	100	50
		Pretreatment	Raw Data- all pretreatment	Raw Data- all pretreatment	SNV- MSC
		Total accuracy	100	100	80
		R2 -RMSEC	98–0.09	98–0.09	98–0.09
	12 water absorbance bands	Healthy	100	100	100 0
		Ulcerative Colitis	100	100	66.6 100
		Crohn's Disease	100	100	50 50
		Pretreatment	Raw Data- all pretreatment	Raw Data- all pretreatment	SNV- MSC Raw Data-Smoothed- Normalized
		Total accuracy	100	100	70 70
		R2 -RMSEC	98–0.09	98–0.09	98–0.09 98–0.09

### Quadratic discrimination analysis (QDA)

Another type of discriminant analysis is Quadratic Discriminant Analysis (QDA). When each group's variability does not have the same structure (unequal covariance matrix) and the curve shape separating groups is not linear, QDA provides a better classification model^14^.

The QDA method in differentiating the plasma samples of the healthy group and the IBD group, using raw data in internal validation, reached 100% total accuracy, sensitivity, and specificity. In external validation, SNV and MSC pretreatments reached the same results (Table 1).

QDA in the range of 12 water absorbance bands, the separation of plasma samples of the healthy group and patients with IBD in the internal validation by SNV, MSC, and normalized pretreatment reached 100% total accuracy, sensitivity, and specificity. In the external validation, by normalized pretreatment, the total accuracy reached 92%, the sensitivity 100%, and the specificity 66% (Table 1).

The QDA method in differentiating the plasma samples of the healthy group, CD, and UC patients, using raw data and various types of pretreatments in the internal validation, reached 100% total accuracy, sensitivity, and specificity. This separation in external validation in SNV and MSC pretreatments with a total accuracy of 84.6% was able to distinguish healthy and UC samples with 100% sensitivity, but it was not able to distinguish CDs due to the small number of CD samples in the calibration and test category. As a result, in the external validation of plasma samples, 100% specificity and 100% sensitivity were achieved in the diagnosis of UC. QDA in the range of 12 water absorbance bands, the separation of plasma samples of the healthy group and CD and UC patients in the internal validation by SNV pretreatment reached 100% total accuracy, sensitivity, and specificity. In the external validation, by SNV and MSC pretreatment, the total accuracy reached 84.6%, the sensitivity was 100%, and the specificity was 0%. These results like the total range of 1300–1600 nm show the important role of water absorbance bands in the detection of groups (Table 1).

In saliva samples, QDA separates the healthy group and IBD patient and then the healthy group, CD, and UC patient samples by SNV and MSC pretreatment, in internal validation with 100% total accuracy, sensitivity, and accuracy. In external validation, the healthy and IBD patients and the healthy and UC patients separate with total accuracy, sensitivity, and specificity of 100%. QDA in the range of 12 water absorbance bands, the separation of saliva samples of patients with IBD in the internal and external validation reached 100% sensitivity. The separation of saliva samples of the healthy group and CD and UC patients in the internal validation reached 100% total accuracy, sensitivity, and specificity (Table 2).

### Support vector machine analysis (SVM)

Support Vector Machine (SVM) is a supervised method that finds an optimal hyperplane or classifier and correctly separates objects into different classes as much as possible. SVM can effectively avoid over-fitting problems by leaving the most significant possible fraction of points from the same group on the same side and maximizing the distance of either group from the hyperplane and structural risk minimum mistake instead of the minimum error of the misclassification on the training set. Due to these advantages, SVM has gained extensive applications, including binary classification^15^.

SVM classification method for raw data and various types of pretreatments of blood plasma samples achieved total accuracy, sensitivity, and specificity of 100% in the healthy group and IBD patient in 1300–1600 nm range (R^2^ = 99%, RMSEC error = 0.04). As a result, the SVM as a supervised method was able to separate healthy, CD, and UC samples with total accuracy, sensitivity, and specificity of 100% (R^2^ = 98%, RMSEC error = 0.09) (Table 1).

For the SVM method in the range of 12 water absorbance bands of blood plasma, the internal and external validation reached a total accuracy, sensitivity, and specificity of 100%. These results show that all the differences between the two groups are manifested in their water absorbance bands (Table 1).

Modeling on the saliva samples of healthy people and IBD patients separated the healthy group from the patients with IBD. The total accuracy, sensitivity, and specificity of 100% were achieved (R^2^ = 99%, RMSEC error = 0.04) (Table 2).

SVM differentiates healthy group, CD, and UC patients with 80% total accuracy, 50% sensitivity in diagnosing CD, 83% sensitivity in diagnosing UC, and 100% specificity in diagnosing healthy samples (R^2^ = 98%, RMSEC error = 0.09). As a result, the SVM supervised method in external validation using saliva samples was able to separate healthy and IBD samples with 100% total accuracy and separate healthy samples from CD and UC patients with 80% total accuracy (Table 2).

In the range of 12 water absorbance bands of saliva, the separation of saliva samples of healthy people and patients with IBD in the internal validation reached 100% total accuracy, sensitivity, and specificity. In the external validation, the total accuracy reached 80%, the sensitivity 100%, and the specificity 0%, which shows that all the effective factors have manifested in their water absorbance bands. SVM in the separation of healthy, CD, and UC patients in the internal validation reached a total accuracy, sensitivity, and specificity of 100%, which was in accordance with the results of the full range of 1300–1600 nm. In the external validation, the results of the water absorbance bands of saliva were responsible for the major part of the separation, and the exact same results were obtained in the raw data and smoothed pretreatment (Table 2).

### Aquagram

The contribution of water absorbance bands was investigated to detecting positive and negative blood plasma or saliva groups separately in IBD disease. The analysis involved examining all wavelengths of blood plasma or saliva samples in the range of 1300–1600 nm (first overtone of water) and specifically focusing on the wavelength of 12 water absorbance bands (WAMACs) in this range. The results of the water absorbance bands were presented using an aquagram based on Eq. (4)^12^.

NIR spectroscopy measurements of WAMACs have been linked to IBD as a bio-perturbation that affects water in the biosystem. In healthy individuals, active water absorbance bands mainly included C1-C4 and C8 bands. However, in IBD patients, these water absorbance bands were significantly reduced, and C11 with 4 hydrogen bonds and C12 with stronger hydrogen bonds were increased.

For UC patients, the mentioned water absorbance bands were greatly reduced. In CD patients, water absorbance bands in the range of C9–C12 were increased, while other bands from C1–C8 were reduced compared to the water absorbance bands of healthy and UC patients. Active water absorbance bands in healthy people mainly included C9–C12 bands. In IBD patients, the mentioned water absorbance bands were greatly reduced, and the C1–C8 water absorbance bands were increased.

**Figure 6:**
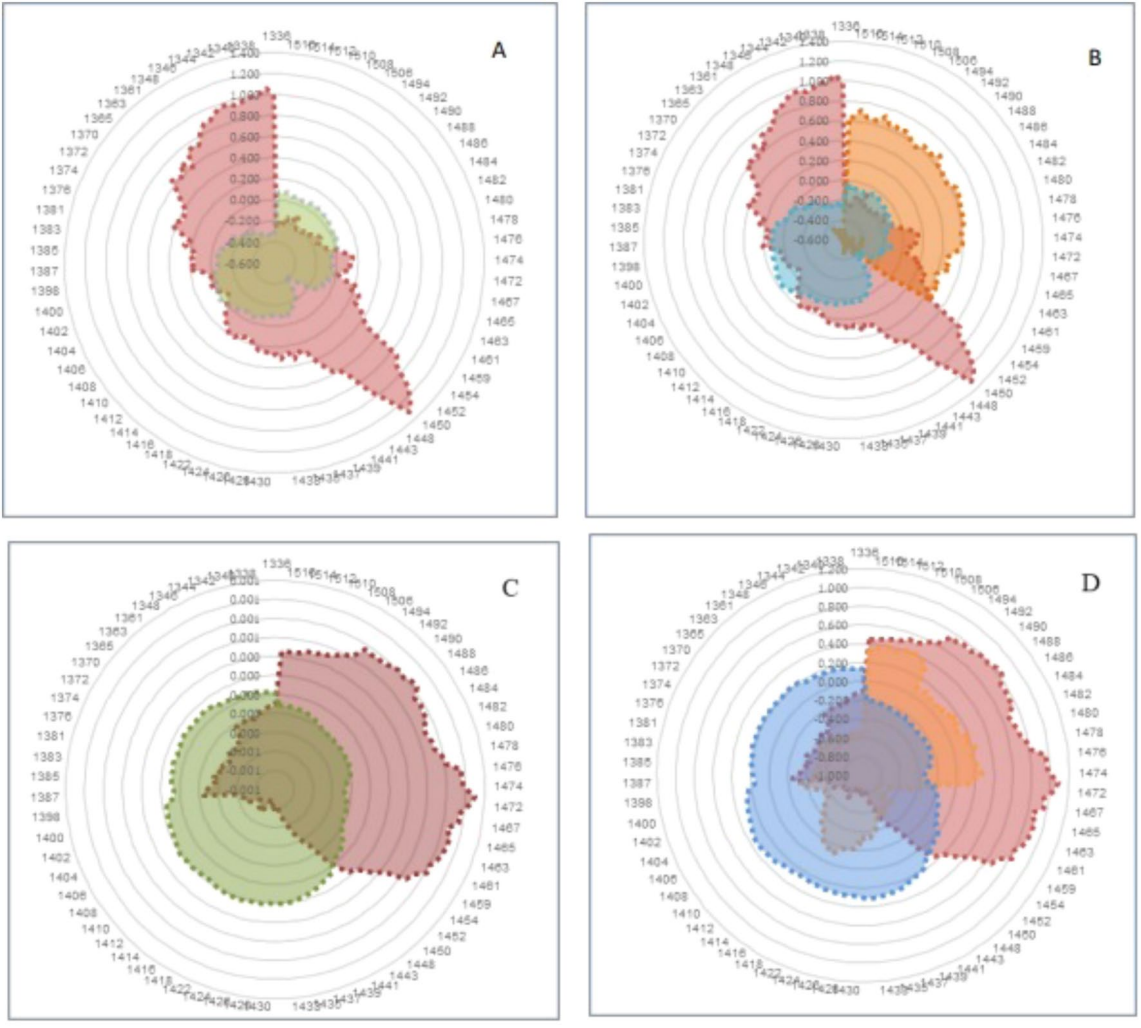
Aquagram. (**A**): blood plasma in IBD patients (Green) and healthy group (Red). (**B**): blood plasma in CD patients (Orange), UC patients (Blue), and healthy group (Red). (**C**): saliva in IBD patients (Green) and healthy group (Red). (**D**): saliva in CD patients (Orange), UC patients (Blue), and the healthy group (Red).

In CD patients, C6 water absorbance band were increased, and C9–C11 water absorbance bands were decreased. For patients with UC, water absorbance bands in the range of C9–C12 were decreased, and C1–C8 bands were increased compared to the water absorbance bands of the healthy group (Fig. 6).

## Discussion

This research includes a new method of diagnosing IBD, including CD and UC, based on the near-infrared spectroscopy method and aquaphotomics approach using blood plasma or saliva samples.

Near-infrared (NIR) spectroscopy is a non-destructive method used to detect functional groups and water bands. It can recognize molecular alterations, understand disease causes, and identify biomarkers. NIR spectroscopy uses regression models to detect substance concentrations and classification models to distinguish between healthy and patient groups.

Aquaphotomics, a new field like genomics and metabolomics, provides valuable insights into biological systems. Water, a comprehensive biomarker, responds to perturbations such as disease, affecting other biomolecules. Using NIR spectroscopy and aquagrams, these changes can be visualized and potentially used as disease biomarkers. Monitoring water's absorbance bands within the 1300–1600 nm range could revolutionize diagnostic methods. This approach offers a non-invasive, rapid, and cost-effective diagnostic method. NIR spectroscopy measurements of WAMACs have been linked to IBD as a bio-perturbation that affects water in the biosystem.

About the effect of MAP and IBD infection, the healthy group and patients were asked about contact with livestock, agricultural activities, non-piped water, non-pasteurized dairy products, family history, place of residence in childhood and now, and occupation. The family history 30%, now resided in cities 70%, spent their childhood years in villages 70%. using piped water now 90%, used non-piped water when they were children 70%, used unpasteurized milk and dairy products during childhood and now50%, were non-slaughterhouse meat consumers 20%, agriculture activity 50%, contact with livestock 40%, in contact with soil and manure 60%, employed in slaughterhouses10% and finally, 80% were in contact with milk and dairy product production. These results can confirm the effect of contact with contaminated livestock and its products, including milk, meat, and their products, and contaminated manure and water on IBD rate.

In raw data, the highest absorption at about 1450 nm confirms that the spectrum of blood plasma and saliva is dominated by water, portion of water in plasma and saliva is 91% and 99%, respectively. In the raw spectrum of the blood plasma, no obvious differences were seen between the healthy group and IBD patients. The saliva average spectrum of IBD patients was higher than the healthy group and CD and UC patients were lower and higher than the average spectrum of healthy people, respectively. Using the average absorbance spectra of the second derivative of the data, the difference between the two groups of healthy and IBD was evident in all wavelengths, especially in the range of 12 water absorbance bands.

The PCA as unsupervised method in blood plasma, by using SNV pretreatment, four principal components described 100% of the variance between the data (PC1, 93%; PC2 with 5%; and PC3 and PC4 with 1% each). In saliva, based on the “Scores” plot by using SNV pretreatment, six PCs described 100% of the variance between the data (PC1 was 84%, PC2 was 10%, PC3 was 3%, and PC4 and PC6 were 1% each). In the space of PC1–PC2, the healthy group and IBD patients, including CDs and UCs, were separated from each other.

Based on the “Loading” plot of PC4 of blood plasma and PC6 of saliva, showed variable wavelengths related to the location of water absorbance bands, which can explain the observed pattern of changes in IBD. By comparing these PCs, more than 12 peaks were identified in the blood plasma “Loading” plot, and these peaks were larger than saliva peaks and had an inverse relationship in most cases. Important wavelengths were observed at 1390, 1402, 1418, 1422, 1434, 1438, 1450, 1485, 1503, 1522, 1537, and 1553 nm.

Based on the above results, all 12 water absorbance bands are active in IBD disease.

The recommended methods to use are the spectroscopic method and aquaphotomics approaches, along with two data mining methods, unsupervised like PCA and supervised methods, to confirm the diagnostic models. We utilized three supervised methods—LDA, QDA, and SVM—to develop diagnostic models. These models were developed twice, once in the full range of 1300–1600 nm and then in the range of 12 water absorbance bands. The developed models are able to distinguish between healthy and diseased samples with 100% accuracy as follows.

In blood plasma, using LDA, the separation of two groups of healthy and patients with IBD reached 100% sensitivity and specificity in internal and external validation. QDA, also as a supervised method, has the same results and then detects the CD and UC patients from the healthy group with total accuracy, sensitivity, and accuracy of 100%. As a result, NIR spectrometry using LDA and QDA models was able to distinguish healthy samples from the IBD patients and its types in the full range (1300–1600 nm).

In saliva samples, using LDA, the separation of two groups of healthy and patients with IBD reached 93.7% sensitivity and 100% specificity in internal validation, and 100% total accuracy, sensitivity, and specificity in external validation. The QDA algorithm achieved 100% overall accuracy, sensitivity, and specificity in the internal validation of the diagnosis of IBD in the first stage and the separation of CD and UC in the second stage. In the external validation of the diagnosis of IBD, the same results were reached, and in the external validation of the diagnosis of CD and UC, it reached 100% specificity and 100% sensitivity in the diagnosis of UC.

In the next step, we used 12 water absorbance bands in the range of 1300–1600 nm of blood plasma and saliva samples. In most cases, the results obtained from the models created with the 12 water absorbance bands were equal to the results of models obtained from the full range of 1300–1600 nm. These results showed that the most effective and dominant factor in the diagnostic power of the obtained models was the effect of water absorbance bands and their changes due to the perturbance factor of the disease and its secondary factors. The important role of water in distinguishing IBD disease is demonstrated by monitoring and evaluating changes between water absorbance bands.

In 2021, LDA model and aquaphotomics approach were used to profile Mannheimia haemolytica infection in dairy calves with accuracy, sensitivity, and specificity > 90%^16^.

The SVM algorithm was used for diagnosing IBD and its types using blood plasma in both internal and external validation, within the range of 1300–1600 nm and 12 water absorbance bands of blood plasma samples. The internal validation of CD and UC achieved a total accuracy, sensitivity, and specificity of 100%. The 12 water absorbance bands made a significant contribution to the diagnostic power of the model.

Similarly, the SVM algorithm was used for diagnosing IBD using saliva in both internal and external validation within the range of 1300–1600 nm and the 12 water absorbance bands. It achieved total accuracy, sensitivity, and specificity of 100% in the internal validation of IBD and its types. In the external validation, the water absorbance bands also had a major contribution to the model's recognition power.

According to the results of the study in 2022, urine Raman spectra as a novel diagnostic tool for CD, with PCA-SVM method and full cross-validation, had 75.5% accuracy in the detection of active and inactive CD^17^.

In 2022, The study using Fourier-Transform Infra-Red (FTIR) microspectroscopy and Partial Least Squares Discriminant Analysis (PLS-DA) to diagnose UC and severity of inflammation reached 91.4% sensitivity and 93.3% specificity to classify normal colon and severe Colitis and used SVM to classify Colitis severity^18^.

The analysis of water absorbance bands provided us with valuable information, and all their wavelengths were used as effective water matrix coordinates (WAMACs) to identify the Water Spectral Patterns of water absorbance bands (WASPs) in blood plasma and saliva samples. These patterns accurately described the health status, disease status, and type of CD or UC. The changes observed in the structure of blood plasma water absorbance bands indicated that in individuals with IBD, the proportion of free water, hydrated water with hydrogen bonds, was significantly reduced. The water molecules were organized in groups of 2 to 4 with more hydrogen bonds, forming stronger bonds between them. In CD patients, the proportion of single-bonded, free, hydrated water molecules decreased, while water molecules with 2-, 3-, and 4-bonds and those with strong bonds increased. In UC patients, the water absorbance bands related to C_1_-C_4_ and C_8_, which included water molecules with symmetric and asymmetric bonds and free water, were greatly reduced compared to healthy samples.

As a result of IBD, in saliva, the share of water molecules with 2 to 4 bonds and strong bonds has decreased and the share of water molecules with one bond, or free water molecules and those with symmetric and asymmetric vibrating bonds has increased. In the saliva of CD patients, the C_6_ water absorbance bands, which include hydrated water increased, the C_9_ water absorbance band decreased sharply, and the C_10_ and C_11_ water absorbance bands also decreased. In patients with UC, water bands in the range of C9 to C_12_ were decreased and C_1_-C_8_ bands were increased compared to the water absorbance bands of healthy people.

The changes in the known water absorbance bands of WAMACs indicate that the structure of water molecules in blood plasma and saliva samples can accurately show the status and type of IBD. WASPs can be a multidimensional biomarker for indicating health status, CD, and UC. By using existing and modern diagnostic methods to create a diagnostic pattern, along with the safety and penetration power of NIR in biological tissues, and the use of a comprehensive biomarker of water that depicts all biochemical changes and secondary substances produced in the body due to disease, it is possible to shorten the diagnosis process and provide a highly accurate diagnosis in minimal time. This can be achieved by using easily accessible blood plasma or saliva samples from patients without the need for sample preparation.

NIR spectroscopy uses blood, serum, plasma, saliva, and tissue to monitor oxygen and Glucose of blood, hemodialysis, oral cancer cells, and many different factors about biomolecules^10^.

In 2017, Colonoscopy-coupled fiber optic probe-based Raman spectroscopy was used as an invasive diagnostic tool with colon biopsy for IBD of the colon (CD and UC) with sparse multinomial logistic regression (SMLR) machine learning algorithm achieved 86.2% sensitivity and 39.7% specificity to IBD, as unique biochemical changes in inflammatory prior to macroscopy changes in tissue, Raman spectroscopy can monitor them^19^.

In 2020, NIR spectroscopy and Aquaphotomics were used to diagnose type 2 Diabetes by the SVM model with 97.2% accuracy, 100% sensitivity, and 95.65% specificity^20^.

The current research presented a novel protocol for the diagnosis of IBD in the first stage and to diagnose the types of this disease, including CD and UC, using NIR spectroscopy and aquaphotomics methods. We applied these methods to diagnosis of Paratuberculosis in dairy cattle by100% Accuracy, sensitivity and specificity^21^.

The aim of current study was to investigate whether the spectra of healthy and patient groups differ from each other. It was found that the major contribution is in water absorbance bands, and models with only water absorbance bands were created. Important biomarkers such as blood plasma and saliva are composed of 90 to 99% water. The results confirm the feasibility of using Near-Infrared spectroscopy to diagnose IBD with 100% accuracy. The active water bands in Inflammatory Bowel Disease were determined, and the separation models based only on the wavelengths related to the active water absorption bands showed significant separation power. Since the changes in water absorbance bands are very precise and comprehensive, in this method, the diagnosis of CD and UCs will not be limited to the diagnosis of the disease agent or its related antibody, and all the processes and metabolites produced in water are shown comprehensively, and it will not overlap or interfere with other diseases.

This non-destructive method using near-infrared spectroscopy and aquaphotomics approach is introduced as a rich information layer next to common diagnostic methods. NIR spectroscopy measurements of WAMACs have been linked to IBD as a bio-perturbation that affects water in the biosystem.

Due to our limited time with the spectrometer in this study, we couldn't analyze more patients. It's important to further investigate this approach with a larger sample size and across different stages of the disease—including silent or active, and mild or severe stages. This will help establish it as a comprehensive diagnostic method, and enable the implementation of appropriate treatment steps to control and manage the disease at different stages.

## Methods

This research was approved by the Research Ethics Committees of Islamic Azad University-Science and Research Branch (SRBIAU)IR.IAU.SRB.REC.1402.391. All experiments were performed according to relevant guidelines and regulations. Informed consent was obtained from all participants.

The study was started on 20 patients referred to the IBD clinic of Isfahan University of Medical Sciences in one day. The patients with missing data were excluded. Finally, ten patients were included (eight UCs and two CDs). Three healthy people were selected as controls. The inclusion criteria were age 20–80 years old and confirmation of the diagnosis based on the European Crohn's and Colitis Organization (ECCO) guideline^22^.

Blood samples were collected in two 5-ml tubes with Ethylenediaminetetraacetic acid (EDTA). After centrifugation at 4000 rpm for 20 min, plasma was separated and stored in a third of 2 ml at − 23 °C until NIR spectroscopy analysis. Saliva samples were collected in sterile containers of 30 ml. Three microtubes with 2 ml of saliva were prepared and stored at − 23 °C for NIR spectroscopy.

### NIR spectral signature collection

Blood plasma and saliva NIR absorbance spectra were collected using a spectrophotometer (UV–VIS-NIR 3600, Shimadzu CO. Japan) equipped with a quartz cuvette having a 1-mm optical path length (n = 39). The samples were thawed over ice for 15 min and warmed between hands for approximately 1 min before NIR spectra collection. NIR spectrum acquired in the range of 1280–1630 nm (interval = 0.5 nm; single scan; very slow). Before collecting plasma and saliva spectra, a reference spectrum was captured from two empty cuvettes, followed by one empty cuvette and one containing distilled water. Three independent spectral signatures were collected per sample, with the cuvette being repacked with plasma or saliva between each replicate.

### Multivariate analysis (MVA)

The chemometrics-based multivariate analysis was performed on the first overtone region of the near-infrared spectrum in the vibrational combination bands between 1300 nm and 1600 nm using Unscrambler X v.10.5. The mathematical pretreatments of the linear baseline correction; standard normal variate (SNV) with detrending polynomial order and a first derivative (symmetric Savitzky–Golay Smoothed, points = 12); smoothed, normalized, multiplicative scatter correction (MSC); and spectroscopic (absorbance to transmittance) are applied to all the databases described next.

A balanced dataset was created by spectral signatures for each category (healthy, or negative, patient, or positive). This dataset contains spectra from all ten patients and three healthy people and was used to perform principal component analysis (PCA), discriminant analysis: LDA, QDA, and SVM, followed by aquaphotomics analyses. In the supervised analysis, datasets were created by positive and negative groups to test mathematical preprocessing and modeling bias against the null hypothesis (no biological signature can differentiate between samples from two classes). The samples were randomly divided into two subsets: a calibration subset for the internal validation set (75%) and a test subset for the external validation set (25%).

### Principal component analysis (PCA)

Principal component analysis (PCA) is an unsupervised Multivariate Analysis and a well-known statistical method for reducing the dimensionality of data sets, explaining variation in data by ignoring the data label, assisting in pattern detecting in spectral behavior, and finding excluded data^14^. The PCA was applied to the dataset, and the calibration sets were created for the discriminant analysis and completed as the first step to observe spectral features from both negative and positive blood plasma and saliva samples to determine dataset groupings and scores distributions, identify dominant peaks in the loadings, and detect outliers using the Hotelling's T2 influence plot.

### Discriminant analysis

Discriminant analysis is a supervised and qualitative classification method that can classify new and unknown samples based on separate models for each group. It also helps to interpret differences between groups using Linear Discriminant Analysis (LDA), and nonlinear discriminant analysis like Quadratic Discriminant Analysis (QDA) methods. LDA is a common technique considering both within-group and between-group variance, decreasing the dimensionality of data increases the variance between and reduces the variance within classes. QDA applies when the variability of each group does not have the same structure (unequal covariance matrix), and the shape of the curve separating groups is not linear^15^.

LDA was used in the raw data, transformed spectra in the 1300–1600 nm range, and then separately for 12 water bonds. Before applying LDA for classifying spectra into positive and negative IBD classes, the dimensionality of each spectral dataset was reduced using PCA to overcome the constraint of requiring more objects (samples) and features (scores or PCs). PCs that captured more than 99% of the variance in the calibration dataset were selected for building the PCA-LDA model. LDA identifies similar spectral features for intra-class grouping and differential spectral features to separate healthy and patient blood plasma or saliva classes. It is reported that the PCA-LDA models from the confusion matrix evaluate the classification method as a percent (%) to describe the quality parameters of accuracy, sensitivity, and specificity.

The PCA-QDA method was then used to describe the nonlinear relationship between groups in raw data and transformed spectra in 1300–1600 nm and 12 water absorbance bands separately.

### Support vector machine (SVM) analysis

Support Vector Machine (SVM) is a supervised method that finds an optimal hyperplane or classifier and correctly separates objects into different classes as much as possible. SVM can effectively avoid over-fitting problems by leaving the most significant possible fraction of points from the same group on the same side and maximizing the distance of either group from the hyperplane and structural risk minimum mistake instead of the minimum error of the misclassification on the training set. Due to these advantages, SVM has gained extensive applications, including binary classification^15^.

LDA, QDA, and SVM, supervised methods were used to achieve the desired prediction models. To evaluate the predictive power of the model obtained from two internal validation methods in total data and calibration data which included 75% of the data “Full validation—leave one out” method was used. The “external validation” method was used on the remaining 25% of the data.

In blood plasma and saliva samples, using the PCA algorithm as an unsupervised method, the dispersion and classification of data in the new space with the presence of 4 main components was completely determined. In order to increase the accuracy in the diagnosis and separation of samples based on the predetermined classification model, the LDA algorithm was used. Then, this process was repeated using the QDA algorithm to find a non-linear relationship between the data with different mean and variance. QDA algorithm was used to diagnose two healthy and IBD patient groups first and then three healthy individuals, patients with CD and UC. We got help from the SVM algorithm as an accurately supervised method for diagnosing IBD and its types using plasma and saliva in internal and external validation.

### Aquaphotomics

In the next step, for Aquaphotomics approach, multivariate analysis by PCA, LDA, QDA, and SVM repeated only for 12 water absorbance bands in the range of 1300–1600 nm of plasma and saliva samples. As the activation of each water absorbance band seems to be different with each perturbation and each sample, Water Matrix Coordinates (WAMACs) are used to present the information about the influence of perturbation on the 12 water absorbance bands in the NIR range for various biological samples, like blood plasma and saliva. A star chart, aquagram, visualized the 12 water absorbance bands changes in Blood plasma and saliva^23^ in reply to IBD perturbation and its types. The 12 water absorbance bands in the 1300–1600 nm range were characterized as follows; C1, 1336–1348, (2ν_3_:H_2_O asymmetric stretching vibration), C2, 1360–1366, (OH–·(H_2_O)_1,2,4_: Water solvation shell), C3, 1370–1376 (ν_1_ + ν_3_: H_2_O symmetrical stretching vibration and H_2_O, asymmetric stretching vibration ), C4, 1380–1388 (OH–·(H_2_O)_1,4_: Water solvation shell, O_2_−·(H_2_O)_4_: Hydrated superoxide clusters, 2ν_1_: H_2_O symmetrical stretching vibration), C5, 1398–1418, (Water confined in a local field of ions (trapped water), S_0_: Free water, Water with free OH–), C6, 1421–1430 (Water hydration band, H-OH bend and O–H⋯O), C7, 1432–1444, (S_1_: Water molecules with 1 hydrogen bond), C8, 1448–1454, (OH-·(H_2_O)_4,5_: Water solvation shell), C9, 1458–1468, (S_2_: Water molecules with 2 hydrogen bonds, 2ν_2_ + ν_3_: H_2_O bending and asymmetrical stretching vibration), C10, 1472–1482, (S_3_: Water molecules with 3 hydrogen bonds), C11, 1482–1495, (S_4_: Water molecules with 4 hydrogen bonds), C12, 1506–1516, (ν_1_: H_2_O symmetrical stretching vibration, ν_2_: H_2_O bending vibration, Strongly bond water)^13^.

### Evaluation of classification methods

Quality parameters, such as accuracy, sensitivity, and specificity, were used to evaluate the classification method's performance. The sensitivity test quantifies the model's ability to correctly identify true positives of IBD, CD, and UC diseases described by Eq. (1), where TP represents the true positive, and FN represents the false negative. A high sensitivity (> 90%) is required when using the prediction model to identify severe but treatable diseases.

The model's specificity shows its ability to identify healthy samples correctly. True negative was shown by Eq. (2), where TN represents true negative, and FP represents false positive. Also, the total accuracy is demonstrated by Eq. (3).

1 $$Sensitivity\text{\% }= (TP/TP + FN) *100$$

2 $$Specificity\text{\% }= (TN/TN+ FP) *100$$

3 $$Total\, accuracy\text{\% }= (((TN*(TN/TN+FP)) + (TP*(TP/TP+FN)))/TN+FP+TP+FN)*100$$

Finally, the water absorbance bands study results are shown by aquagram using Eq. (4)^12^. Where A' λ is the normalized absorbance value displayed on the radar axis; Aλ is absorbance after scatter correction (multiplicative scatter correction using the mean of the dataset as a reference spectrum or standard normal variant transformation); µλ is the mean of all spectral; σλ is the standard deviation of all spectral; and λ are the selected wavelengths from WAMACS regions corresponding to the activated water absorbance bands^12^.

4 $${A}^{\prime}\lambda =(A\lambda - \mu \lambda )/\sigma \lambda$$

### Questionnaire

About the effect of MAP and the IBD infection, the healthy group and patients were asked about contact with livestock, agricultural activities, non-piped water, non-pasteurized dairy products, family history, place of residence in childhood and now, and occupation.

## Data Availability

Data is provided within the manuscript.
