# Author Correction: Non-destructive diagnosis of Inflammatory Bowel Disease by near-infrared spectroscopy and aquaphotomics

**DOI:** 10.1038/s41598-024-74707-8

**Published:** 2024-10-04

**Authors:** Saba Behdad, Reza Massudi, Abbas Pakdel

**Affiliations:** 1 Department of Animal Science, College of Agriculture, Science and Research Branch, Islamic Azad University, Tehran, 14778-93855 Iran; 2 Laser and Plasma Research Institute, Shahid Beheshti University, Tehran, 19839-69411 Iran; 3 Department of Animal Science, College of Agriculture, Isfahan University of Technology (IUT), Isfahan, 84156-83111 Iran

Correction to: *Scientific Reports*10.1038/s41598-024-65443-0, published online 10 July 2024

The original version of this Article contained an error in Affiliation 3, which was incorrectly given as ‘Department of Animal Science, Isfahan University of Technology, Isfahan, 84156-83111, Iran’.

The correct affiliation is listed below.

Department of Animal Science, College of Agriculture, Isfahan University of Technology (IUT), Isfahan 84156-83111, Iran.

The original Article has been corrected.

